# Supplementary material for: Efficient and Stable Air-Processed Ternary Organic Solar Cells Incorporating Gallium-Porphyrin as an Electron Cascade Material
Source: Nanomaterials (Basel). 2023 Oct 21;13(20):2800. doi: 10.3390/nano13202800 (PMC10609146; doi:10.3390/nano13202800)
Supplement: Supplementary file 1 [file nanomaterials-13-02800-s001.zip › nanomaterials-2567815-supplementary.pdf]

Supplementary Materials

Efficient and Stable Air-Processed Ternary Organic Solar Cells Incorporating Gallium-Porphyrin as an Electron Cascade Material

Anastasia Soultati [1], Maria Verouti [1,2], Ermioni Polydorou [1], Konstantina-Kalliopi Armadorou [1], Zoi Georgiopoulou [1,3], Leonidas C. Palilis [2], Ioannis Karatasios [1], Vassilis Kilikoglou [1], Alexander Chroneos [4,5], Athanassios G. Coutsolelos [6,*], Panagiotis Argitis [1,*] and Maria Vasilopoulou [1,*]

[1] Institute of Nanoscience and Nanotechnology (INN), National Center for Scientific Research (NCSR) Demokritos, 15341 Agia Paraskevi, Greece
[2] Department of Physics, University of Patras, 26504 Rio Patra, Greece
[3] Solid State Physics Section, Physics Department, National and Kapodistrian University of Athens, Panepistimioupolis, 15784 Athens, Greece
[4] Department of Electrical and Computer Engineering, University of Thessaly, 38221 Volos, Greece
[5] Department of Materials, Imperial College, London SW7 2AZ, UK
[6] Laboratory of Bioinorganic Chemistry, Department of Chemistry, University of Crete, Voutes Campus, P.O. Box 2208, 71003 Heraklion, Greece
* Correspondence: acoutsol@uoc.gr (A.G.C.); p.argitis@inn.demokritos.gr (P.A.); m.vasilopoulou@inn.demokritos.gr (M.V.)

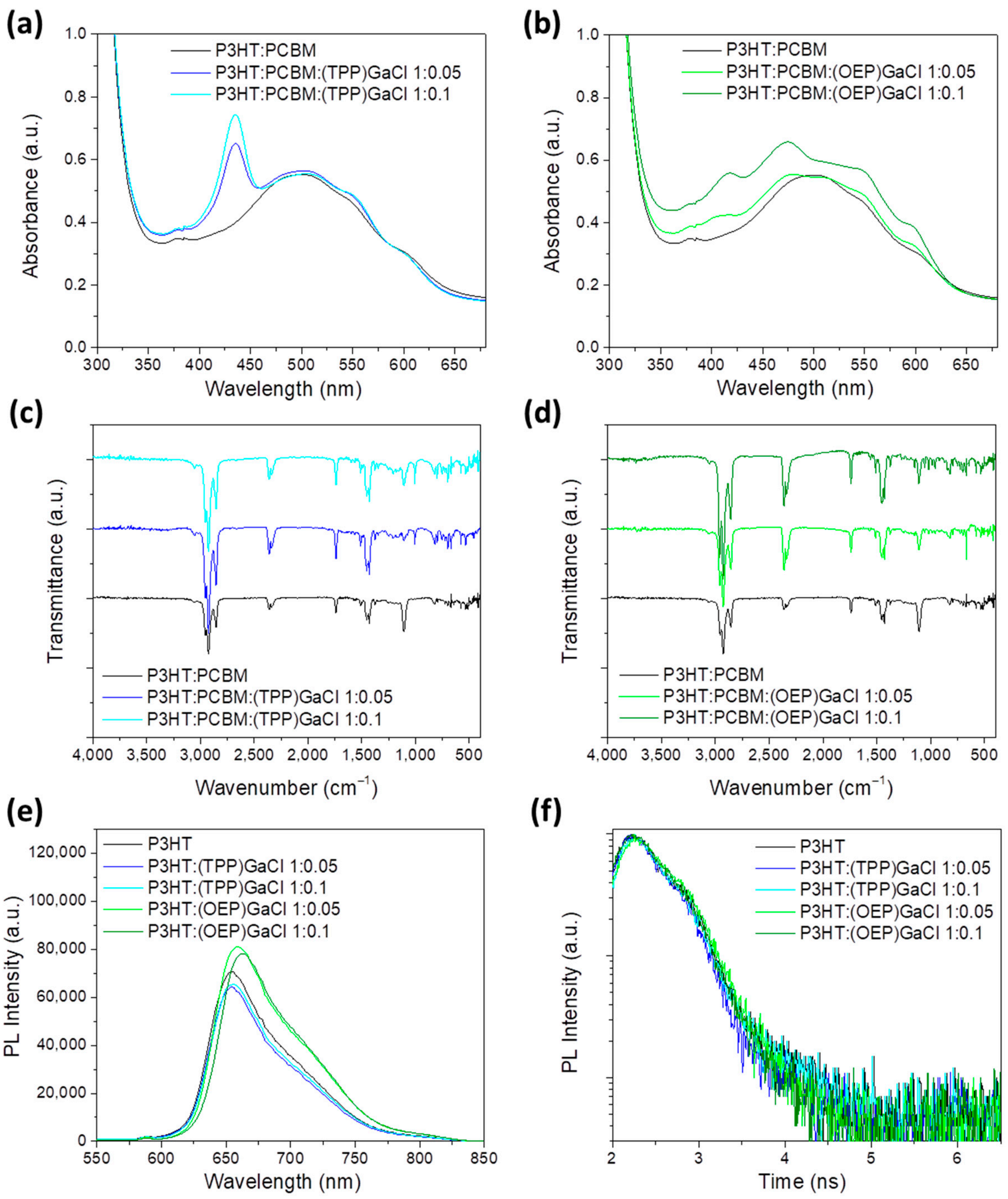

Figure S1. UV-Vis absorption spectra of binary and ternary P3HT:PCBM films with porphyrin additives: (a) (TPP)GaCl and (b) (OEP)GaCl in various ratios. FTIR spectra of binary and ternary P3HT:PCBM films with porphyrin additives: (c) (TPP)GaCl and (d) (OEP)GaCl in various ratios. (e) Steady-state PL spectra of P3HT without or with (TPP)GaCl and (OEP)GaCl in various ratios and (f) Transient PL dynamics of the same samples.

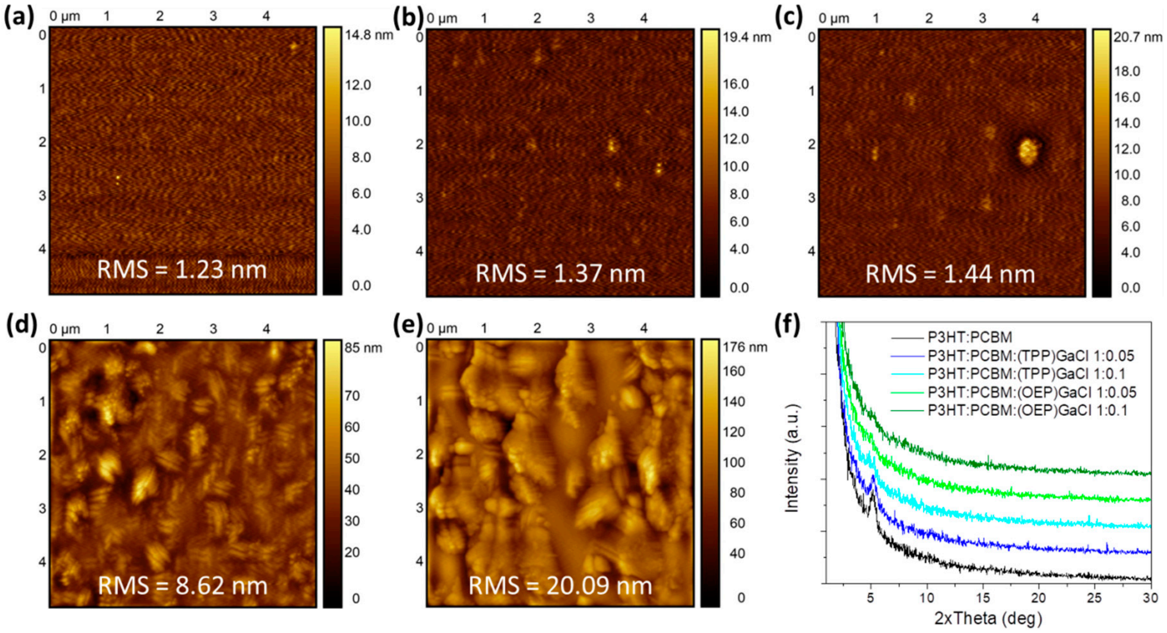

Figure S2. 5 × 5 μm^2 AFM height images of (a) binary P3HT:PCBM, (b) P3HT:PCBM:(TPP)GaCl 1:0.05 (c) P3HT:PCBM:(TPP)GaCl 1:0.1, (d) P3HT:PCBM:(OEP)GaCl 1:0.05 and (e) P3HT:PCBM:(OEP)GaCl 1:0.1 ternary blended films. (f) XRD patterns of the same samples.

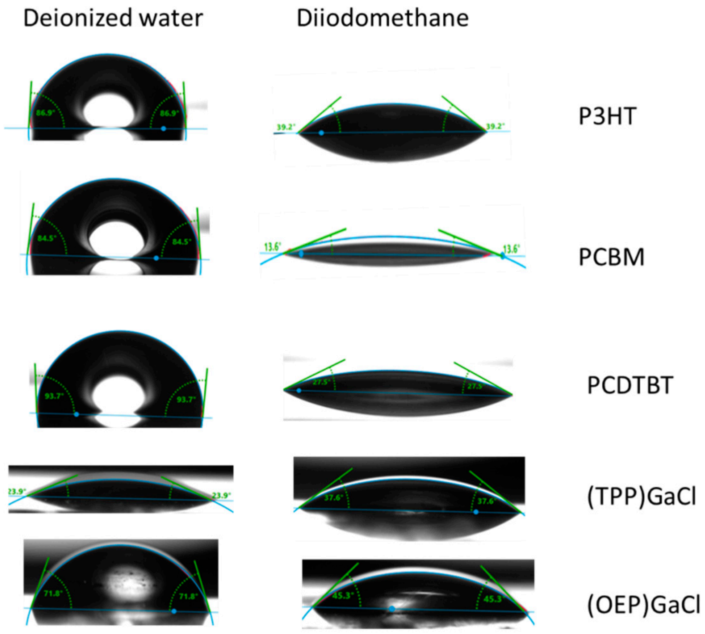

Figure S3. Contact angle measurements of deionized water and diiodomethane droplets on P3HT, PCBM, PCDTBT, (TPP)GaCl, and (OEP)GaCl films deposited on glass substrates.

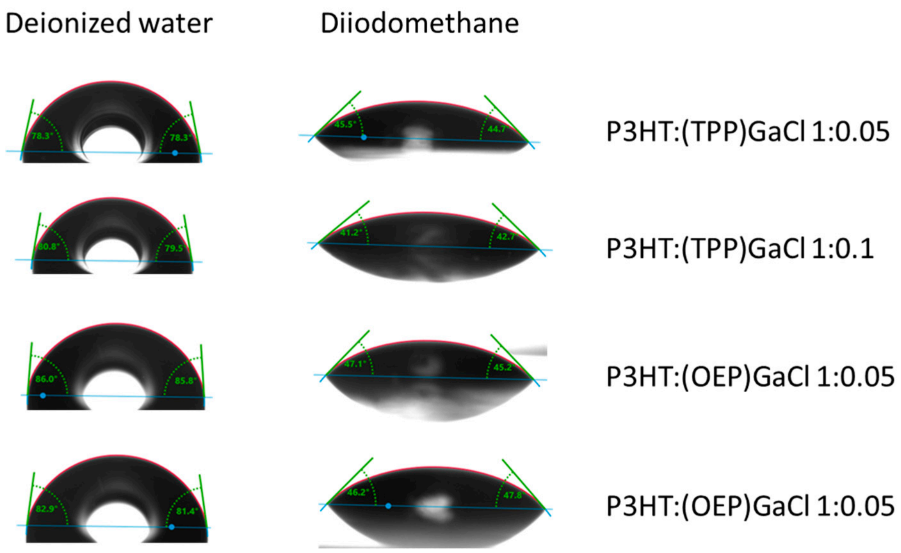

Figure S4. Contact angle measurements of deionized water and diiodomethane droplets on binary P3HT:(TPP)GaCl and P3HT:(OEP)GaCl films deposited on glass substrates.

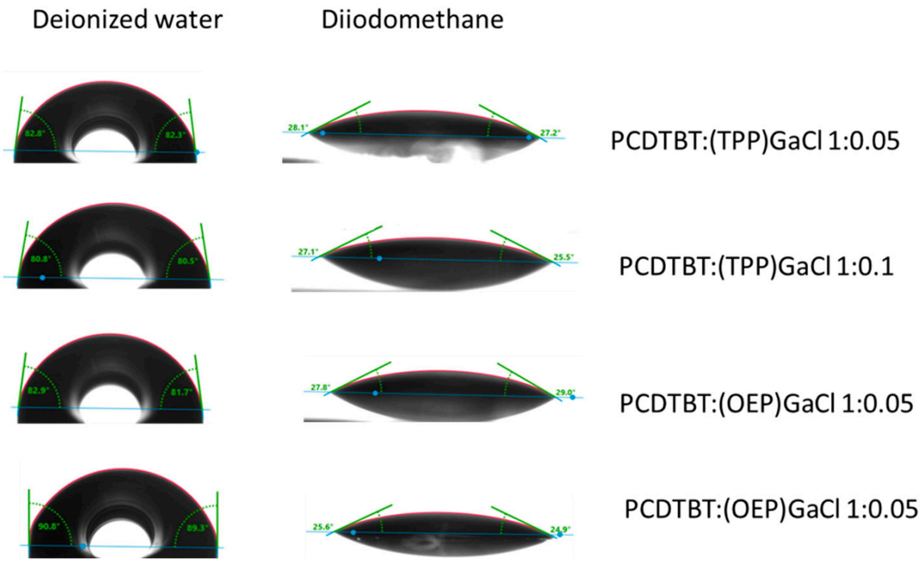

Figure S5. Contact angle measurements of deionized water and diiodomethane droplets on binary PCDTBT:(TPP)GaCl and PCDTBT:(OEP)GaCl films deposited on glass substrates.

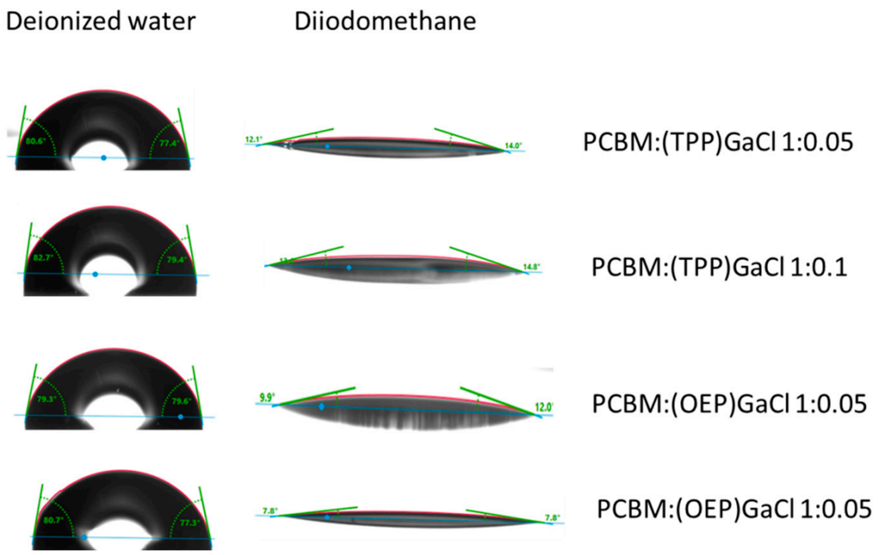

Figure S6. Contact angle measurements of deionized water and diiodomethane droplets on binary PCBM:(TPP)GaCl and PCBM:(OEP)GaCl films deposited on glass substrates.

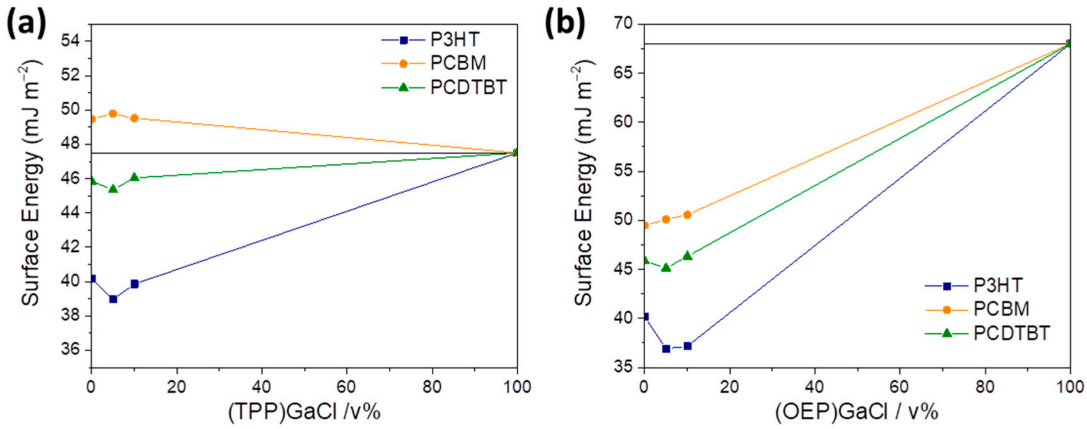

Figure S7. Surface energy of (a) P3HT:(TPP)GaCl, PCDTBT:(TPP)GaCl, and PCBM:(TPP)GaCl binary films, and (b) P3HT:(OEP)GaCl, PCDTBT:(OEP)GaCl, and PCBM:(OEP)GaCl binary films. The solid black line represents the surface of the neat porphyrin.

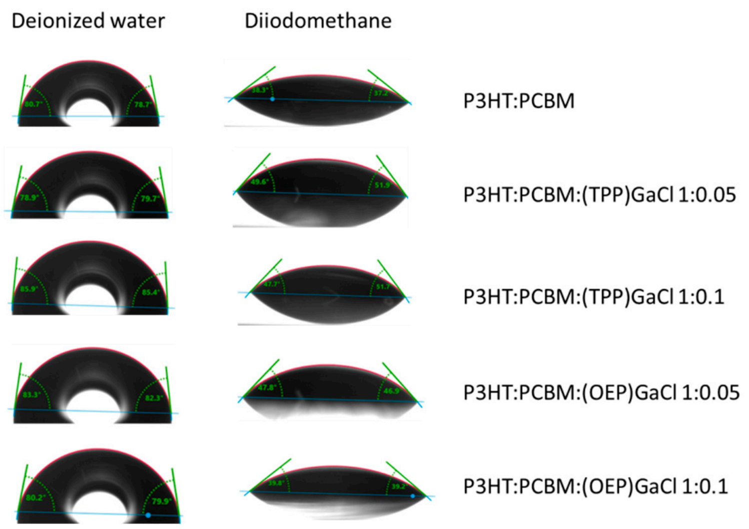

Figure S8 Contact angle measurements of deionized water and diiodomethane droplets on binary P3HT:PCBM and ternary P3HT:PCBM:porphyrin-GaCl films deposited on glass substrates.

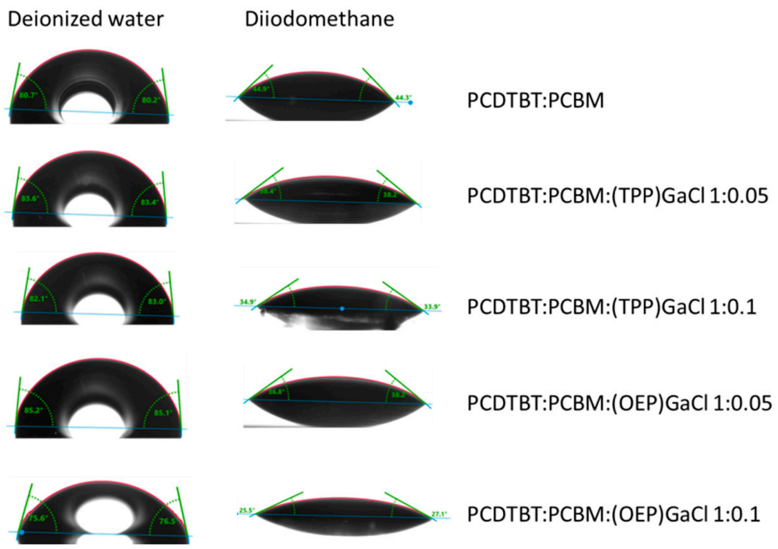

Figure S9 Contact angle measurements of deionized water and diiodomethane droplets on binary PCDTBT:PCBM and ternary PCDTBT:PCBM:porphyrin-GaCl films deposited on glass substrates.

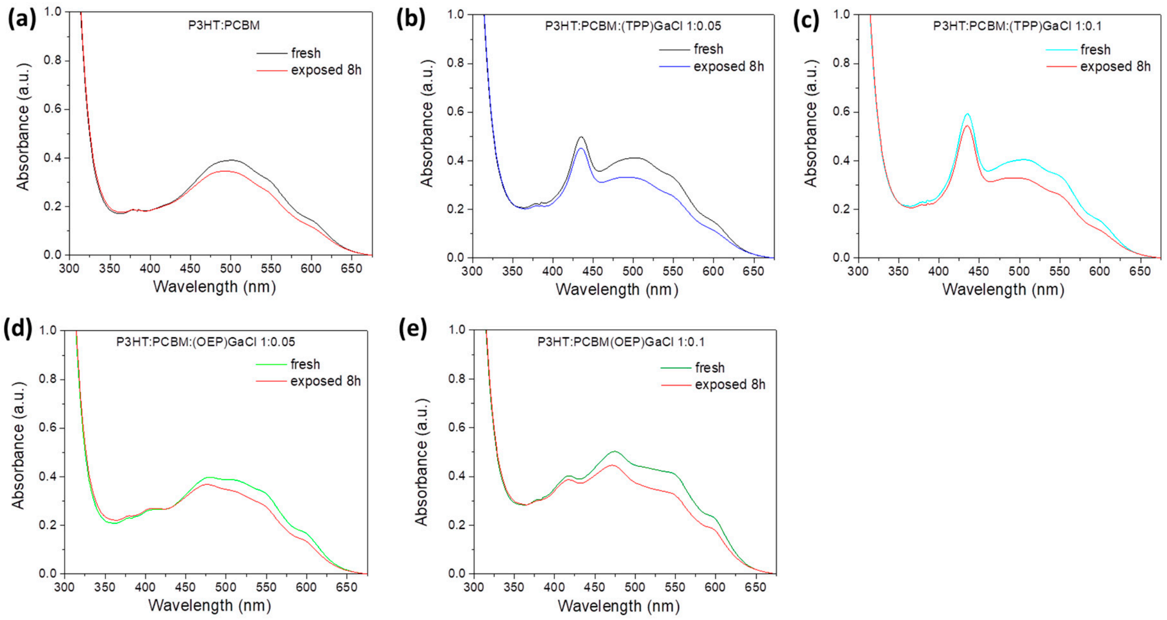

Figure S10 UV-Vis absorption spectra of fresh and exposed to sunlight illumination for 8 h of (a) binary P3HT:PCBM and ternary (b) P3HT:PCBM:(TTP)GaCl 1:0.05, (c) P3HT:PCBM:(TTP)GaCl 1:0.1, (d) P3HT:PCBM:(OEP)GaCl 1:0.05, and (e) P3HT:PCBM:(OEP)GaCl 1:0.1 films.

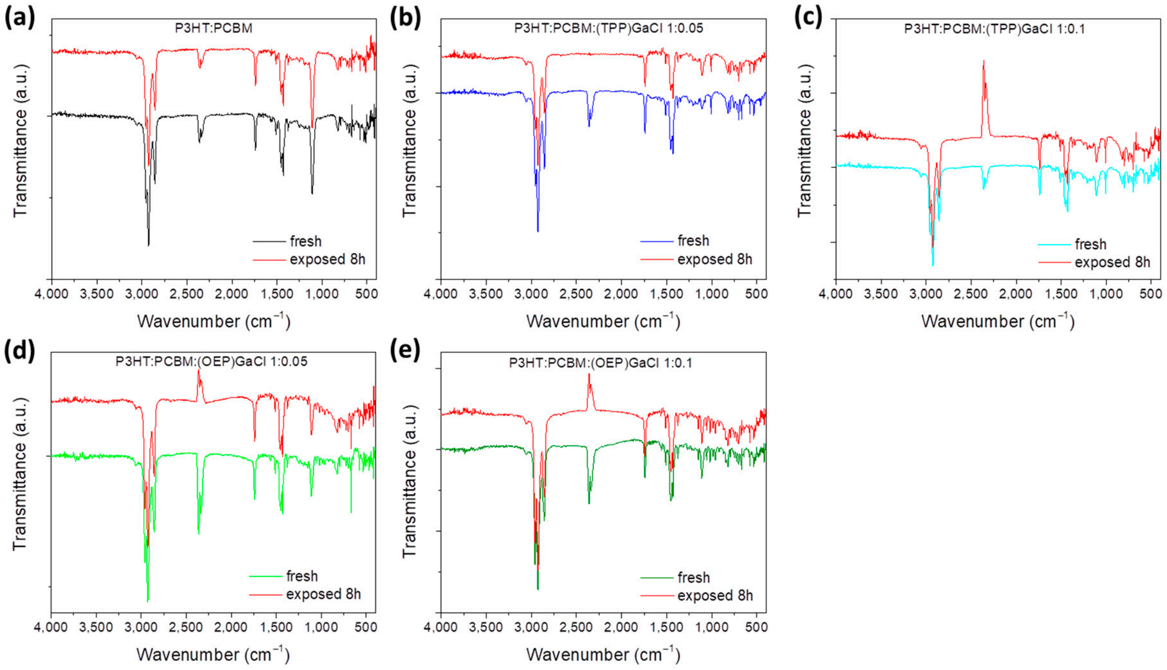

Figure S11 FTIR spectra of fresh and exposed to sunlight illumination for 8 h of (a) binary P3HT:PCBM and ternary (b) P3HT:PCBM:(TTP)GaCl 1:0.05, (c) P3HT:PCBM:(TTP)GaCl 1:0.1, (d) P3HT:PCBM:(OEP)GaCl 1:0.05, and (e) P3HT:PCBM:(OEP)GaCl 1:0.1 films.

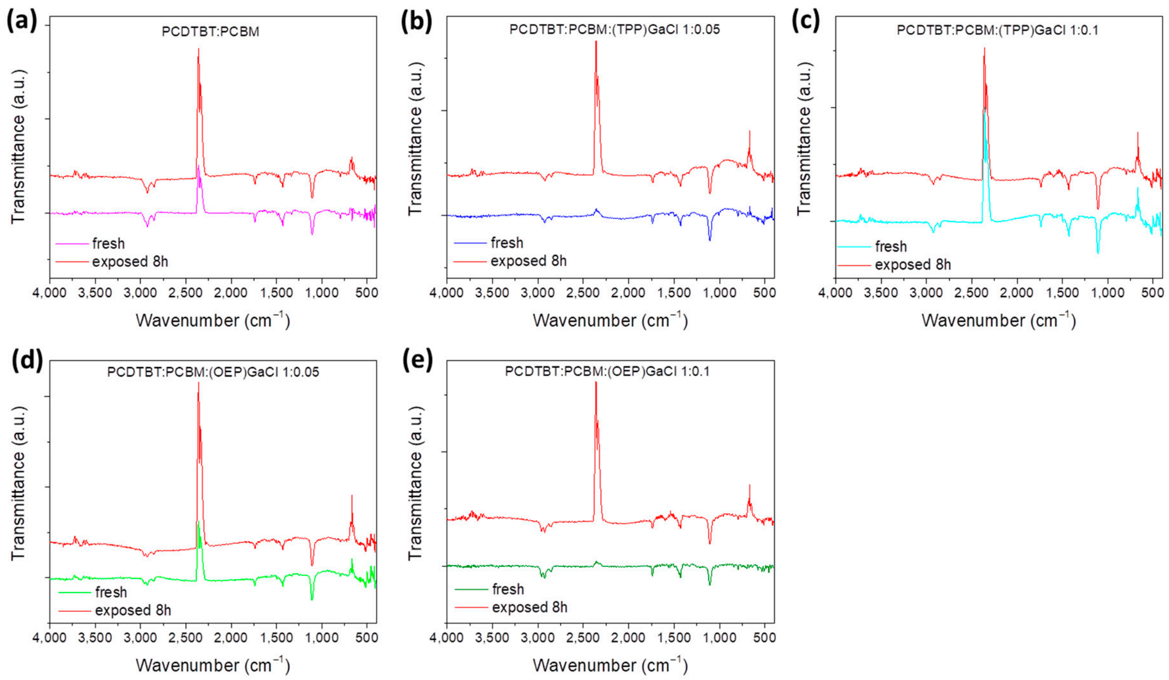

Figure S12 FTIR spectra of fresh and exposed to sunlight illumination for 8 h of (a) binary PCDTBT:PCBM and ternary (b) PCDTBT:PCBM:(TTP)GaCl 1:0.05, (c) PCDTBT:PCBM:(TTP)GaCl 1:0.1, (d) PCDTBT:PCBM:(OEP)GaCl 1:0.05, and (e) PCDTBT:PCBM:(OEP)GaCl 1:0.1 films.

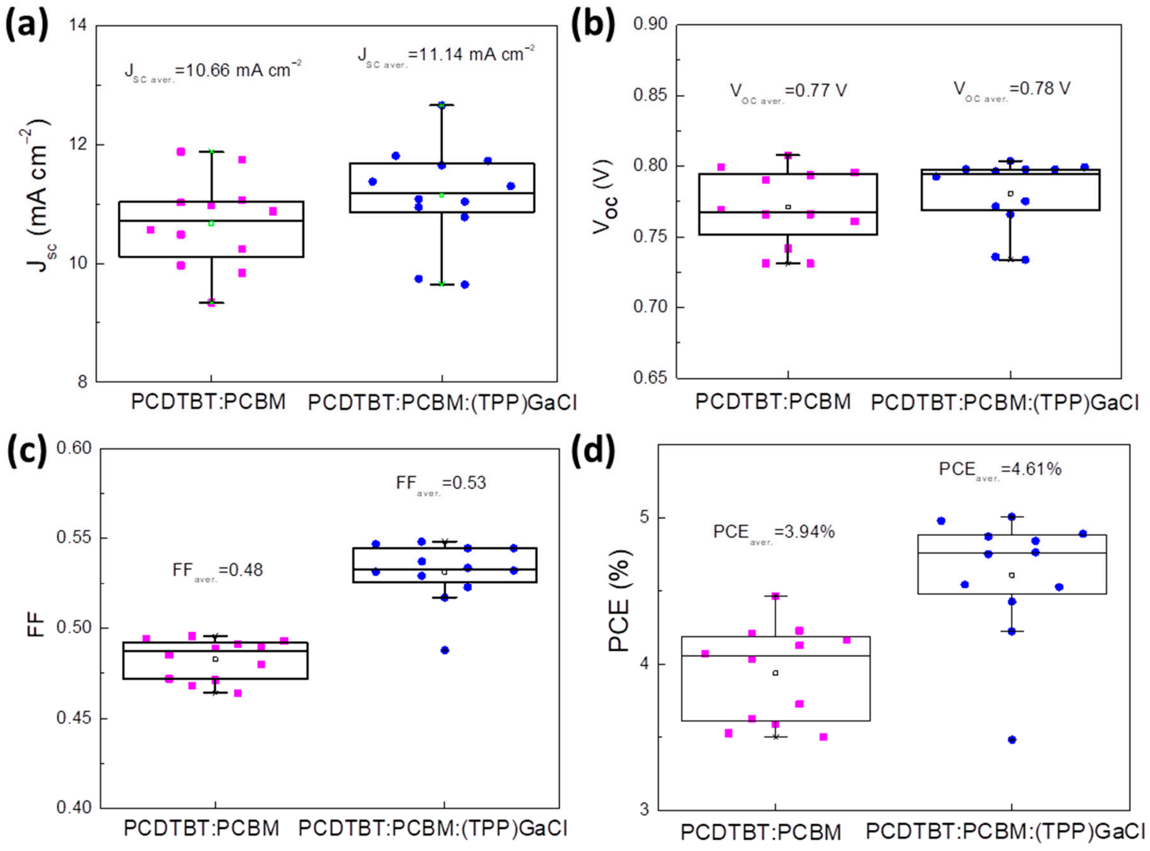

Figure S13 Statistical analysis of the performance parameters of PCDTBT:PCBM and PCDTBT:PCBM:(TPP)GaCl OSCs (12 devices for each type): (a) Jsc, (b) Voc, (c) FF and (d) PCE.

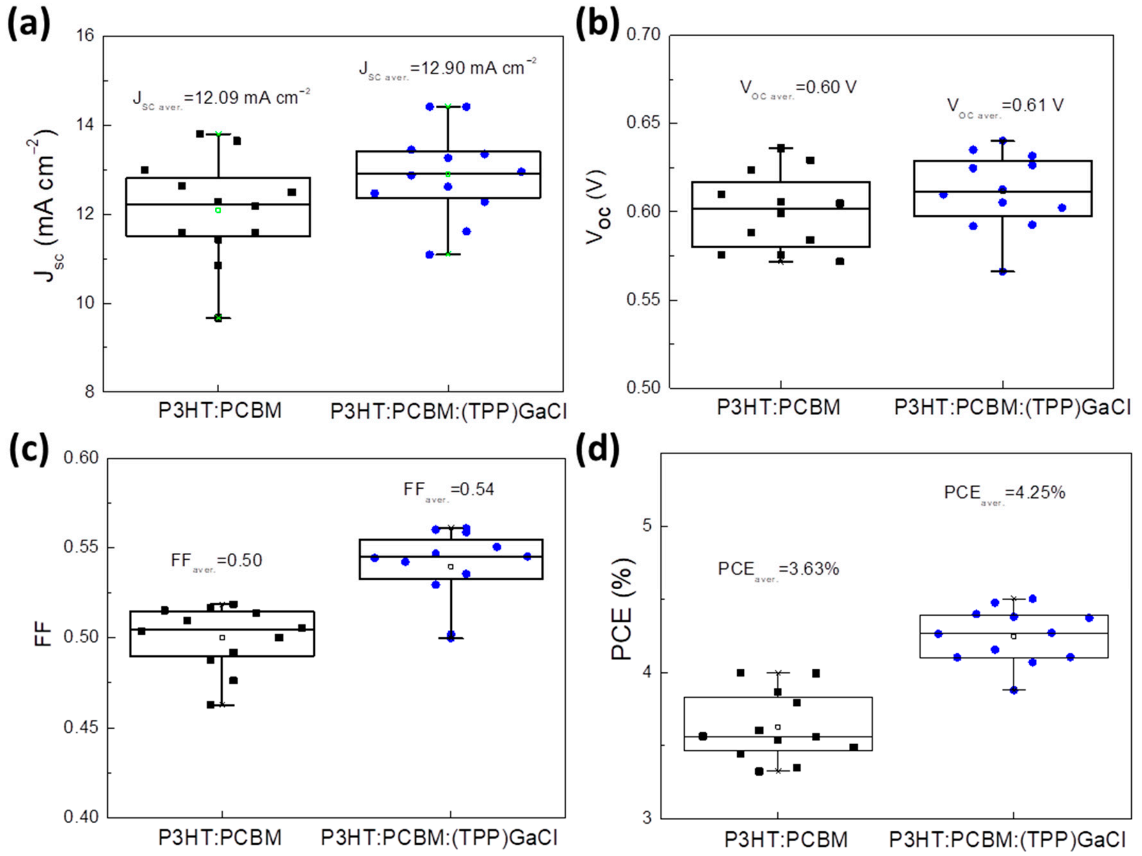

Figure S14 Statistical analysis of the performance parameters of PCDTBT:PCBM and P3HT:PCBM:(TPP)GaCl OSCs (12 devices for each type): (a) Jsc, (b) Voc, (c) FF and (d) PCE.

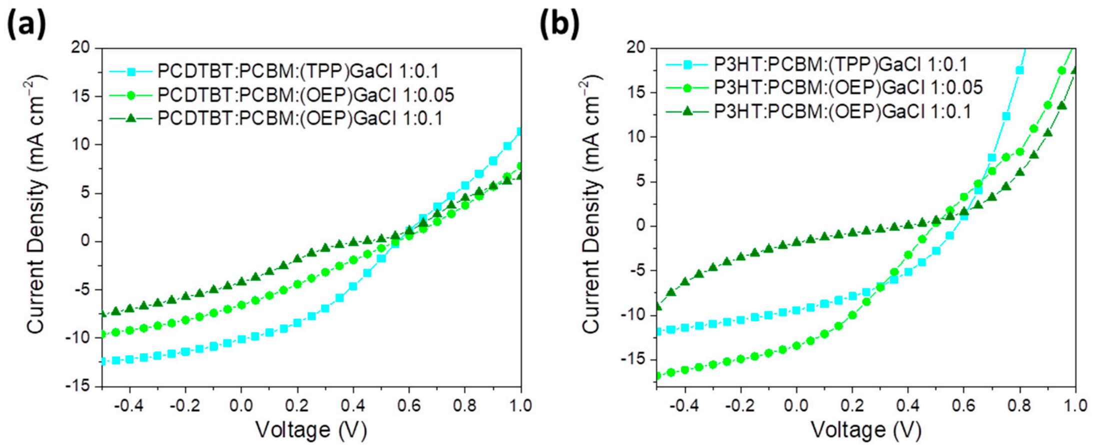

Figure S15 J-V characteristic curves under 1.5 AM illumination of ternary OSCs based on (a) PCDTBT:PCBM and (b) P3HT:PCBM with (TPP)GaCl 1:0.1, (OEP)GaCl 1:0.05 and (OEP)GaCl 1:0.1.

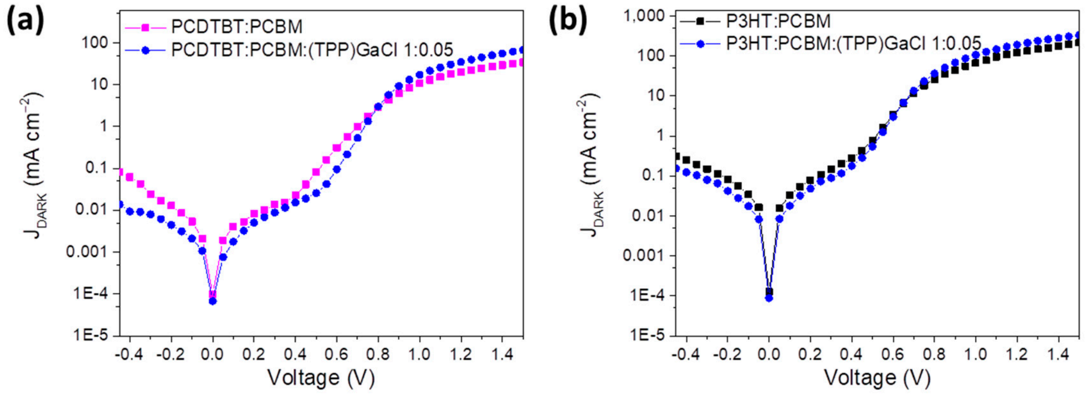

Figure S16 Dark J-V characteristics curves of the binary and ternary OSCs based on (a) PCDTBT:PCBM and (b) P3HT:PCBM blends.

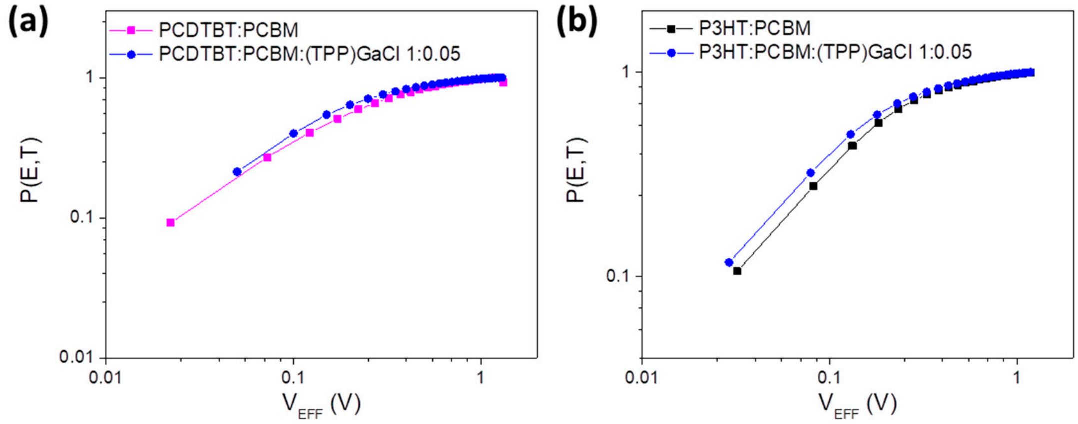

Figure S17 Exciton dissociation probability (P(E,T)) versus the effective voltage (V_{EFF}) of binary and ternary OSCs based on (a) PCDTBT:PCBM and (b) P3HT:PCBM blends.

Table S1 Contact angle and surface energy of various films.

**Materials**	**Contact Angle (°)**	**Surface Energy (mN m^{-1})**
P3HT	86.9	40.2
PCBM	84.5	49.5
PCDTBT	93.7	45.7
(OEP)GaCl	23.9	68.1
(TPP)GaCl	71.8	41.0

Table S2 Wetting coefficient of (TPP)GaCl and (OEP)GaCl added in P3HT:PCBM and PCDTBT:PCBM blends.

**Blends**	**Ratio**	**Wetting coefficient**
P3HT:PCBM:(TPP)GaCl	1:0.05	0.26
P3HT:PCBM:(TPP)GaCl	1:01	0.23
P3HT:PCBM:(OEP)GaCl	1:0.05	0.32
P3HT:PCBM:(OEP)GaCl	1:0.1	0.32
PCDTBT:PCBM:(TPP)GaCl	1:0.05	0.11
PCDTBT:PCBM:(TPP)GaCl	1:01	0.09
PCDTBT:PCBM:(OEP)GaCl	1:0.05	0.13
PCDTBT:PCBM:(OEP)GaCl	1:0.1	0.11
